# 2-Methyl-*N*-[(3-methyl-2-pyrid­yl)carbamothio­yl]benzamide

**DOI:** 10.1107/S1600536808009513

**Published:** 2008-04-10

**Authors:** B. M. Yamin, S. Yousuf, M. S. M. Yusof, R. H. Jusoh

**Affiliations:** a School of Chemical Sciences and Food Technology, Universiti Kebangsaan Malaysia, UKM 43500 Bangi Selangor, Malaysia; b HEJ Research Institute of Chemistry, International Center for Chemical and Biological Sciences, University of Karachi, Karachi 75270, Pakistan; c Department of Chemistry, Universiti Malaysia Terengganu, Manngabang Telipot, Terengganu, Malaysia

## Abstract

In the title compound, C_15_H_15_N_3_OS, the thio­urea group is stabilized by an intra­molecular hydrogen bond between the carbonyl O atom and the thio­amide group. A C—H⋯N intramolecular hydrogen bond is also present. Mol­ecules are linked by inter­molecular N—H⋯O and C—H⋯S hydrogen bonds.

## Related literature

For the crystal structure of *N*-(3-iodo­phen­yl)-*N*′-(2-methyl­benzo­yl)thio­urea, see: Yusof *et al.* (2007 ▶). For bond-length data, see: Allen *et al.* (1987 ▶).
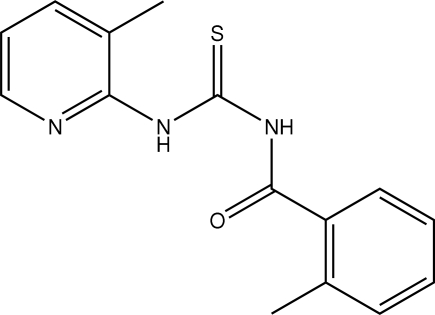


## Experimental

### 

#### Crystal data

C_15_H_15_N_3_OS
*M* *_r_* = 285.36
Monoclinic, 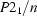

*a* = 7.955 (3) Å
*b* = 7.811 (3) Å
*c* = 23.414 (8) Å
β = 90.827 (6)°
*V* = 1454.6 (9) Å^3^
*Z* = 4
Mo *K*α radiation
μ = 0.22 mm^−1^
*T* = 298 (2) K
0.49 × 0.46 × 0.17 mm

#### Data collection

Bruker SMART APEX CCD area-detector diffractometer
Absorption correction: multi-scan (*SADABS*; Bruker, 2000 ▶) *T*_min_ = 0.899, *T*_max_ = 0.963
7524 measured reflections
2710 independent reflections
2099 reflections with *I* > 2σ(*I*)
*R*_int_ = 0.018

#### Refinement

*R*[*F* ^2^ > 2σ(*F* ^2^)] = 0.041
*wR*(*F* ^2^) = 0.112
*S* = 1.02
2710 reflections
191 parameters
1 restraint
H atoms treated by a mixture of independent and constrained refinement
Δρ_max_ = 0.24 e Å^−3^
Δρ_min_ = −0.13 e Å^−3^

### 

Data collection: *SMART* (Bruker, 2000 ▶); cell refinement: *SAINT* (Bruker, 2000 ▶); data reduction: *SAINT*; program(s) used to solve structure: *SHELXS97* (Sheldrick, 2008 ▶); program(s) used to refine structure: *SHELXL97* (Sheldrick, 2008 ▶); molecular graphics: *SHELXTL* (Sheldrick, 2008 ▶); software used to prepare material for publication: *SHELXTL*, *PARST* (Nardelli, 1995 ▶) and *PLATON* (Spek, 2003 ▶).

## Supplementary Material

Crystal structure: contains datablocks global, I. DOI: 10.1107/S1600536808009513/sg2229sup1.cif

Structure factors: contains datablocks I. DOI: 10.1107/S1600536808009513/sg2229Isup2.hkl

Additional supplementary materials:  crystallographic information; 3D view; checkCIF report

## Figures and Tables

**Table 1:** Hydrogen-bond geometry (Å, °)

*D*—H⋯*A*	*D*—H	H⋯*A*	*D*⋯*A*	*D*—H⋯*A*
N2—H2⋯O1	0.86 (2)	2.04 (2)	2.697 (2)	132.2 (18)
C15—H15*A*⋯N2	0.96	2.56	2.961	105
N2—H2⋯O1^i^	0.86 (2)	2.30 (2)	3.021 (2)	142 (2)
C13—H13⋯S1^ii^	0.93	2.85	3.700	154

Symmetry codes: (i) 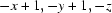; (ii) 
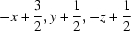
.

**Fig. 1.**
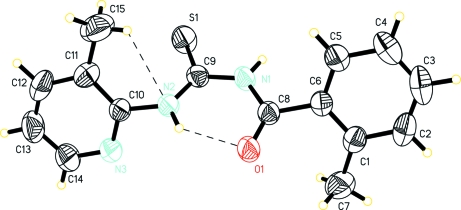
The molecular Structure of (1) with displacement ellipsoids drawn at 50% probability level.The dashed lines indicates the intramolecular hydrogen bonds.

**Fig. 2.**
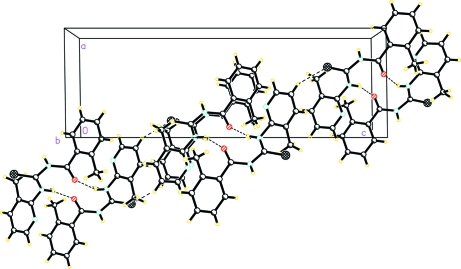
A packing diagram of (1). Hydrogen bonds are shown by dashed lines.

## supplementary crystallographic information

### Comment

The title compound, (I), is analogous to *N*-(3-iodophenyl)-*N*'-(2-methylbenzoyl) thiourea (II), (Yusof *et al.*, 2007) except that the iodophenyl group is replaced by the 3-methylpyridine group (Fig.1). The bond lengths and angles are in normal range (Allen *et al.*, 1987). The central thiourea moiety, S1/N1/N2/C9, pyridine, N3/(C10—C14), and benzene,(C1—C6) rings are each planar with maximum deviation of 0.033 (2)Å for N2 atom from the least square plane. The central thiourea moiety makes dihedral angle with the pyridine and benzene rings of 64.58 (8) and 62.03 (8)° respectively. The dihedral angle between the pyridine and benzene rings (4.03 (10)°) is smaller compared to that in (II) of 31.88 (9)°. The molecule maintains the *trans*-*cis* geometry of the thiourea moiety which is stabilized by the intrahydrogen bond between the carbonyl oxygen atom O1 and the thioamide hydrogen atom, H15A. In the crystal structure, the molecules are linked by the N2—H2···O1 and C13—H13···S1 intermolecular hydrogen bonds (symmtery codes as in Table 2).

### Experimental

The mixture of 2-methylbenzoyl chloride (9.720 g, 0.025mole) with the equimolar amount of ammonium thiocyanate (1.903 g, 0.025 mol) and 2-amino-3-methyl pyridine,(2.703 g, 0.025 mol) in 40 ml dry acetone was refluxed with stirring for 4 h. The solution was filtered and left to evaporate at room temperature. The colourless crystals obtained after a few days, was found suitable for X-ray investigations. The yield was 85% and the melting point is 412.3–413.8 K.

### Refinement

H atoms on the C of methyl, phenyl and pyridine were positioned geomatrically with C—H=0.96 Å and 0.93 Å respectively and constrained to ride on their parent atoms with *U*_iso_(H)= 1.2*U*_eq_(CH) and 1.5*U*_eq_(CH_3_). The hydrogen atoms attached to the amino nitrogen atoms were located from the difference Fourier map and refined isotropically.

### Crystal data

**Table:**

C_15_H_15_N_3_OS	*F*_000_ = 600
*M**_r_* = 285.36	*D*_x_ = 1.303 Mg m^−^^3^
Monoclinic, *P*2_1_/*n*	Mo *K*α radiation λ = 0.71073 Å
Hall symbol: -P 2yn	Cell parameters from 2292 reflections
*a* = 7.955 (3) Å	θ = 1.7–25.5º
*b* = 7.811 (3) Å	µ = 0.22 mm^−^^1^
*c* = 23.414 (8) Å	*T* = 298 (2) K
β = 90.827 (6)º	Block, colourless
*V* = 1454.6 (9) Å^3^	0.49 × 0.46 × 0.17 mm
*Z* = 4	

### Data collection

**Table:**

Bruker SMART APEX CCD area-detector diffractometer	2710 independent reflections
Radiation source: fine-focus sealed tube	2099 reflections with *I* > 2σ(*I*)
Monochromator: graphite	*R*_int_ = 0.019
Detector resolution: 83.66 pixels mm^-1^	θ_max_ = 25.5º
*T* = 298(2) K	θ_min_ = 1.7º
ω scans	*h* = −9→9
Absorption correction: multi-scan(SADABS; Bruker, 2000)	*k* = −9→9
*T*_min_ = 0.899, *T*_max_ = 0.963	*l* = −16→28
7524 measured reflections	

### Refinement

**Table:**

Refinement on *F*^2^	Secondary atom site location: difference Fourier map
Least-squares matrix: full	Hydrogen site location: inferred from neighbouring sites
*R*[*F*^2^ > 2σ(*F*^2^)] = 0.041	H atoms treated by a mixture of independent and constrained refinement
*wR*(*F*^2^) = 0.112	*w* = 1/[σ^2^(*F*_o_^2^) + (0.0591*P*)^2^ + 0.3029*P*] where *P* = (*F*_o_^2^ + 2*F*_c_^2^)/3
*S* = 1.02	(Δ/σ)_max_ < 0.001
2710 reflections	Δρ_max_ = 0.24 e Å^−^^3^
191 parameters	Δρ_min_ = −0.13 e Å^−^^3^
1 restraint	Extinction correction: none
Primary atom site location: structure-invariant direct methods	

### Special details

**Table:**

Geometry. All e.s.d.'s (except the e.s.d. in the dihedral angle between two l.s. planes) are estimated using the full covariance matrix. The cell e.s.d.'s are taken into account individually in the estimation of e.s.d.'s in distances, angles and torsion angles; correlations between e.s.d.'s in cell parameters are only used when they are defined by crystal symmetry. An approximate (isotropic) treatment of cell e.s.d.'s is used for estimating e.s.d.'s involving l.s. planes.
Refinement. Refinement of *F*^2^ against ALL reflections. The weighted *R*-factor *wR* and goodness of fit *S* are based on *F*^2^, conventional *R*-factors *R* are based on *F*, with *F* set to zero for negative *F*^2^. The threshold expression of *F*^2^ > σ(*F*^2^) is used only for calculating *R*-factors(gt) *etc*. and is not relevant to the choice of reflections for refinement. *R*-factors based on *F*^2^ are statistically about twice as large as those based on *F*, and *R*- factors based on ALL data will be even larger.

### Fractional atomic coordinates and isotropic or equivalent isotropic displacement parameters (Å^2^)

**Table:**

	*x*	*y*	*z*	*U*_iso_*/*U*_eq_	
S1	0.34243 (7)	0.20705 (8)	0.17685 (2)	0.0662 (2)	
O1	0.40058 (17)	0.34898 (19)	−0.00872 (5)	0.0599 (4)	
N1	0.26335 (19)	0.2529 (2)	0.06915 (6)	0.0485 (4)	
H1	0.1847 (19)	0.183 (2)	0.0784 (8)	0.056 (6)*	
N2	0.49690 (18)	0.4025 (2)	0.10075 (6)	0.0460 (4)	
H2	0.513 (2)	0.429 (3)	0.0656 (9)	0.057 (6)*	
N3	0.77881 (19)	0.3995 (2)	0.12320 (7)	0.0579 (4)	
C1	0.1548 (2)	0.1298 (2)	−0.07366 (8)	0.0497 (5)	
C2	0.0123 (3)	0.0935 (3)	−0.10668 (9)	0.0641 (6)	
H2A	0.0238	0.0305	−0.1401	0.077*	
C3	−0.1439 (3)	0.1477 (3)	−0.09150 (10)	0.0707 (7)	
H3	−0.2358	0.1239	−0.1152	0.085*	
C4	−0.1669 (3)	0.2364 (3)	−0.04197 (11)	0.0675 (6)	
H4	−0.2737	0.2720	−0.0316	0.081*	
C5	−0.0286 (2)	0.2727 (3)	−0.00736 (9)	0.0537 (5)	
H5	−0.0430	0.3303	0.0270	0.064*	
C6	0.1310 (2)	0.2234 (2)	−0.02376 (7)	0.0434 (4)	
C7	0.3230 (3)	0.0627 (3)	−0.09092 (10)	0.0759 (7)	
H7A	0.3091	−0.0141	−0.1226	0.114*	
H7B	0.3742	0.0028	−0.0594	0.114*	
H7C	0.3936	0.1565	−0.1018	0.114*	
C8	0.2781 (2)	0.2805 (2)	0.01149 (7)	0.0433 (4)	
C9	0.3749 (2)	0.2940 (2)	0.11353 (7)	0.0457 (4)	
C10	0.6291 (2)	0.4528 (2)	0.13916 (7)	0.0437 (4)	
C11	0.6000 (3)	0.5570 (3)	0.18580 (8)	0.0554 (5)	
C12	0.7424 (3)	0.5988 (3)	0.21814 (9)	0.0688 (6)	
H12	0.7315	0.6667	0.2505	0.083*	
C13	0.8968 (3)	0.5421 (3)	0.20313 (10)	0.0745 (7)	
H13	0.9914	0.5683	0.2252	0.089*	
C14	0.9096 (3)	0.4468 (3)	0.15529 (10)	0.0732 (7)	
H14	1.0162	0.4123	0.1442	0.088*	
C15	0.4302 (3)	0.6260 (4)	0.20004 (11)	0.0852 (8)	
H15A	0.3581	0.6201	0.1669	0.128*	
H15B	0.4408	0.7430	0.2121	0.128*	
H15C	0.3829	0.5593	0.2303	0.128*	

### Atomic displacement parameters (Å^2^)

**Table:**

	*U*^11^	*U*^22^	*U*^33^	*U*^12^	*U*^13^	*U*^23^
S1	0.0669 (4)	0.0892 (4)	0.0423 (3)	−0.0180 (3)	−0.0071 (2)	0.0199 (3)
O1	0.0565 (8)	0.0822 (10)	0.0411 (7)	−0.0295 (7)	0.0001 (6)	0.0024 (7)
N1	0.0422 (9)	0.0642 (10)	0.0390 (8)	−0.0158 (7)	−0.0032 (6)	0.0087 (7)
N2	0.0425 (8)	0.0628 (10)	0.0325 (8)	−0.0091 (7)	−0.0020 (6)	0.0037 (7)
N3	0.0422 (9)	0.0730 (11)	0.0585 (10)	−0.0011 (8)	−0.0012 (7)	−0.0153 (8)
C1	0.0598 (12)	0.0452 (10)	0.0441 (10)	−0.0082 (9)	−0.0028 (8)	0.0020 (8)
C2	0.0840 (16)	0.0569 (13)	0.0508 (11)	−0.0154 (12)	−0.0169 (11)	−0.0021 (10)
C3	0.0697 (15)	0.0612 (13)	0.0801 (16)	−0.0157 (11)	−0.0364 (13)	0.0138 (12)
C4	0.0452 (12)	0.0651 (14)	0.0919 (17)	−0.0024 (10)	−0.0117 (11)	0.0112 (13)
C5	0.0485 (12)	0.0559 (12)	0.0567 (12)	−0.0043 (9)	−0.0023 (9)	0.0011 (9)
C6	0.0455 (10)	0.0432 (9)	0.0413 (9)	−0.0070 (8)	−0.0044 (7)	0.0061 (8)
C7	0.0810 (16)	0.0757 (16)	0.0713 (15)	0.0002 (13)	0.0119 (12)	−0.0179 (12)
C8	0.0434 (10)	0.0456 (10)	0.0408 (9)	−0.0068 (8)	−0.0005 (7)	0.0018 (8)
C9	0.0387 (10)	0.0565 (11)	0.0418 (10)	−0.0005 (8)	−0.0014 (7)	0.0026 (8)
C10	0.0435 (10)	0.0508 (10)	0.0367 (9)	−0.0025 (8)	−0.0028 (7)	0.0003 (8)
C11	0.0634 (12)	0.0592 (12)	0.0435 (10)	0.0039 (10)	0.0008 (9)	−0.0035 (9)
C12	0.0903 (17)	0.0698 (15)	0.0459 (11)	−0.0074 (13)	−0.0106 (11)	−0.0148 (10)
C13	0.0637 (15)	0.0915 (18)	0.0675 (14)	−0.0146 (13)	−0.0233 (11)	−0.0064 (13)
C14	0.0435 (12)	0.0973 (18)	0.0785 (15)	−0.0010 (11)	−0.0096 (10)	−0.0162 (13)
C15	0.0838 (17)	0.0948 (18)	0.0774 (16)	0.0215 (14)	0.0110 (13)	−0.0214 (14)

### Geometric parameters (Å, °)

**Table:**

S1—C9	1.6545 (18)	C4—H4	0.9300
O1—C8	1.214 (2)	C5—C6	1.386 (3)
N1—C8	1.374 (2)	C5—H5	0.9300
N1—C9	1.394 (2)	C6—C8	1.490 (2)
N1—H1	0.860 (9)	C7—H7A	0.9600
N2—C9	1.326 (2)	C7—H7B	0.9600
N2—C10	1.429 (2)	C7—H7C	0.9600
N2—H2	0.86 (2)	C10—C11	1.384 (3)
N3—C10	1.321 (2)	C11—C12	1.392 (3)
N3—C14	1.327 (3)	C11—C15	1.497 (3)
C1—C2	1.392 (3)	C12—C13	1.357 (3)
C1—C6	1.394 (3)	C12—H12	0.9300
C1—C7	1.498 (3)	C13—C14	1.350 (3)
C2—C3	1.364 (3)	C13—H13	0.9300
C2—H2A	0.9300	C14—H14	0.9300
C3—C4	1.366 (3)	C15—H15A	0.9600
C3—H3	0.9300	C15—H15B	0.9600
C4—C5	1.386 (3)	C15—H15C	0.9600
			
C8—N1—C9	129.35 (15)	H7A—C7—H7C	109.5
C8—N1—H1	114.8 (13)	H7B—C7—H7C	109.5
C9—N1—H1	114.6 (13)	O1—C8—N1	122.17 (16)
C9—N2—C10	124.63 (15)	O1—C8—C6	122.97 (16)
C9—N2—H2	119.5 (13)	N1—C8—C6	114.85 (14)
C10—N2—H2	114.5 (13)	N2—C9—N1	116.05 (15)
C10—N3—C14	117.05 (18)	N2—C9—S1	126.06 (14)
C2—C1—C6	116.94 (19)	N1—C9—S1	117.88 (13)
C2—C1—C7	120.18 (19)	N3—C10—C11	124.77 (17)
C6—C1—C7	122.84 (18)	N3—C10—N2	113.16 (15)
C3—C2—C1	122.0 (2)	C11—C10—N2	121.92 (16)
C3—C2—H2A	119.0	C10—C11—C12	115.06 (19)
C1—C2—H2A	119.0	C10—C11—C15	123.32 (19)
C2—C3—C4	120.8 (2)	C12—C11—C15	121.6 (2)
C2—C3—H3	119.6	C13—C12—C11	121.0 (2)
C4—C3—H3	119.6	C13—C12—H12	119.5
C3—C4—C5	119.1 (2)	C11—C12—H12	119.5
C3—C4—H4	120.5	C14—C13—C12	118.4 (2)
C5—C4—H4	120.5	C14—C13—H13	120.8
C4—C5—C6	120.2 (2)	C12—C13—H13	120.8
C4—C5—H5	119.9	N3—C14—C13	123.7 (2)
C6—C5—H5	119.9	N3—C14—H14	118.1
C5—C6—C1	120.92 (17)	C13—C14—H14	118.1
C5—C6—C8	118.59 (16)	C11—C15—H15A	109.5
C1—C6—C8	120.42 (16)	C11—C15—H15B	109.5
C1—C7—H7A	109.5	H15A—C15—H15B	109.5
C1—C7—H7B	109.5	C11—C15—H15C	109.5
H7A—C7—H7B	109.5	H15A—C15—H15C	109.5
C1—C7—H7C	109.5	H15B—C15—H15C	109.5
			
C6—C1—C2—C3	0.5 (3)	C10—N2—C9—N1	−176.19 (16)
C7—C1—C2—C3	177.9 (2)	C10—N2—C9—S1	4.8 (3)
C1—C2—C3—C4	−1.8 (3)	C8—N1—C9—N2	14.3 (3)
C2—C3—C4—C5	0.7 (3)	C8—N1—C9—S1	−166.69 (16)
C3—C4—C5—C6	1.8 (3)	C14—N3—C10—C11	1.6 (3)
C4—C5—C6—C1	−3.1 (3)	C14—N3—C10—N2	177.29 (19)
C4—C5—C6—C8	173.72 (17)	C9—N2—C10—N3	113.7 (2)
C2—C1—C6—C5	2.0 (3)	C9—N2—C10—C11	−70.4 (3)
C7—C1—C6—C5	−175.40 (18)	N3—C10—C11—C12	−2.8 (3)
C2—C1—C6—C8	−174.81 (17)	N2—C10—C11—C12	−178.13 (18)
C7—C1—C6—C8	7.8 (3)	N3—C10—C11—C15	175.1 (2)
C9—N1—C8—O1	0.3 (3)	N2—C10—C11—C15	−0.2 (3)
C9—N1—C8—C6	−178.45 (18)	C10—C11—C12—C13	1.2 (3)
C5—C6—C8—O1	−129.5 (2)	C15—C11—C12—C13	−176.7 (2)
C1—C6—C8—O1	47.3 (3)	C11—C12—C13—C14	1.3 (4)
C5—C6—C8—N1	49.2 (2)	C10—N3—C14—C13	1.3 (4)
C1—C6—C8—N1	−133.98 (18)	C12—C13—C14—N3	−2.8 (4)

### Hydrogen-bond geometry (Å, °)

**Table:**

*D*—H···*A*	*D*—H	H···*A*	*D*···*A*	*D*—H···*A*
N2—H2···O1	0.86 (2)	2.04 (2)	2.697 (2)	132.2 (18)
C15—H15A···N2	0.96	2.56	2.961	105
N2—H2···O1^i^	0.86 (2)	2.30 (2)	3.021 (2)	142 (2)
C13—H13···S1^ii^	0.93	2.85	3.700	154

Symmetry codes:  (i) −*x*+1, −*y*+1, −*z*; (ii) −*x*+3/2, *y*+1/2, −*z*+1/2.
